# The interaction between flagellin and the glycosphingolipid Gb3 on host cells contributes to *Bacillus cereus* acute infection

**DOI:** 10.1080/21505594.2020.1773077

**Published:** 2020-06-07

**Authors:** Song Gao, Chengpei Ni, Wenhua Huang, Huaijie Hao, Hua Jiang, Qingyu Lv, Yuling Zheng, Peng Liu, Decong Kong, Yongqiang Jiang

**Affiliations:** a State Key Laboratory of Pathogen and Biosecurity, Institute of Microbiology and Epidemiology, Academy of Military Medical Sciences, Beijing, China; b CAS Key Laboratory of Pathogenic Microbiology and Immunology, Institute of Microbiology, Chinese Academy of Sciences, Beijing, China

**Keywords:** *Bacillus cereus*, Gb3, flagellin, adhesion, virulence factor

## Abstract

Bacillus cereus

is an opportunistic pathogen that can cause emetic or diarrheal foodborne illness. Previous studies have identified multiple pathogenic *B. cereus* strains and characterized a variety of virulence factors. Here, we demonstrate that the virulence and lethality of *B. cereus* for mammalian cells and host animals involve the interaction of *B. cereus* flagellin proteins and the host-cell-surface-localized glycosphingolipid Gb3 (CD77, Galα1-4Galβ1-4Glcβ1-Cer). We initially found that *B. cereus* infection was less lethal for Gb3-deficiencient *A4galt*^−/-^ mice than for wild-type mice. Subsequent experiments established that some factor other than secreted toxins must account of the observed differential lethality: Gb3-deficiencient *A4galt*^−/-^ mice were equally susceptible to secreted-virulence-factor-mediated death as WT mice, and we observed no differences in the bacterial loads of spleens or livers of mice treated with *B. cereus* strain vs. mice infected with a mutant variant of incapable of producing many secreted toxins. A screen for host-interacting *B. cereus* cell wall components identified the well-known flagellin protein, and both flagellin knockout strain assays and Gb3 inhibitor studies confirmed that flagellin does interact with Gb3 in a manner that affects *B. cereus* infection of host cells. Finally, we show that treatment with polyclonal antibody against flagellin can protect mice against *B. cereus* infection. Thus, beyond demonstrating a previously unappreciated interaction between a bacterial motor protein and a mammalian cell wall glycosphingolipid, our study will provide useful information for the development of therapies to treat infection of *B. cereus*.

## Introduction

*Bacillus cereus*, a member of the *Bacillus cereus* group, is well known as a foodborne pathogen. The symptoms of food poisoning caused by *B. cereus* include diarrhea and emesis, and these symptoms have been linked to enterotoxins and cereulide [1]. In addition, *B. cereus* also causes local and systemic nongastrointestinal infections in immunocompromised persons as well as immunocompetent individuals [2,3]. Accordingly, the nongastrointestinal infections include fulminant bacteremia, pneumonia, endophthalmitis, central nervous system infections, and respiratory and urinary tract infections [2] *B. cereus* infection can even cause patient death, especially in patients presenting with bacteremia [4].

Globotriaosylceramide (Gb3/CD77, Galα1-4Galβ1-4Glcβ1-Cer) is a subtype of glycosphingolipid that is synthesized by the addition of galactose to lactosylceramide, and this reaction is catalyzed by α-1,4-galactosyltransferase, which is encoded by the *A4GALT* gene [5,6]. Gb3 is mainly expressed on the surface of epithelial or endothelial cells of the intestine, kidneys, and brain [7,8]. Gb3 is a cell-surface receptor, and the Galα1-4Gal (galabiose) moiety is recognized by a number of bacterial virulence factors such as lectin I (LecA) of *Pseudomonas aeruginosa*, Shiga toxins of *Shigella dysenteriae*, and streptococcal adhesin P (SadP) of *Streptococcus suis* [9–11]. Shiga toxins have an AB5 structure, and the pentameric B-subunit is responsible for Gb3 binding. Shiga toxins are internalized into early endosomes after binding of the B-subunit of Shiga toxins to Gb3 [12]. SadP is a cell wall adhesin, and its specific binding to Gb3 contributes to adhesion to human intestinal epithelial cells [13]. Gb3 performs various functions during different pathogen infections; however, the function of Gb3 in *B. cereus* infection remains unclear.

The adhesion of bacteria to the host often facilitates host subversion and colonization during the infection [14]. Prior studies found that *B. cereus* can adhere to epithelial cells, fibronectin and mucin [15,16]. In addition, the adhesion of *B. cereus* to epithelial cells is involved in its virulence [17]. In addition to flagella, The *B. cereus* cell wall peptidase FM (CwpFM) has been described to participate in adhesion, but the receptor for the enzyme is unknown [15,18]. The flagella of bacteria are traditionally considered as only motility organelles, but it has recently been clearly demonstrated that flagella can function as adhesins. Flagella consist of many different proteins, among which flagellin plays a role in adhesion. However, the receptor of flagellin has not been determined for many pathogens [19]. Sánchez et al. revealed that some *B. cereus* surface proteins such as cell-bound proteases, S-layer components, and flagellin bind to mucin or fibronectin, but the functions of these proteins in adhesion have not been verified [16].

In the present work, we demonstrated that Gb3 contributed to the rapid death of mice during *B. cereus* infection. Flagellin was identified to bind to Gb3, and we found that the interaction between flagellin and Gb3 was involved in the adhesion of *B. cereus* to host cells, thereby contributing to *B. cereus* acute infection.

## Materials and methods

### Bacterial strain and growth condition

The wild-type strain *B. cereus* HN001 is a clinical isolate that caused a food poisoning incident in Henan Province, China [20]. Evolutionarily, it is closely related to the pathogenic strain B4264 and FORC_005 in evolution, and it was kindly provided by Professor Hengliang Wang of the Academy of Military Medical Sciences (Beijing, China) [21,22]. *B. cereus* was grown in brain heart infusion (BHI) medium at 37°C, and the mid-log growth phase bacteria were harvested for experimentation. *Escherichia coli* (*E. coli*) was grown in Luria-Bertani (LB) medium and antibiotics were used at concentrations of 50 μg/ml kanamycin, 34 μg/ml chloramphenicol, and 100 μg/ml spectinomycin, in addition, 300 μg/ml spectinomycin for *B. cereus*.

*B. cereus* strains featuring deletion of *fla* (WR52_08290) and *plcR* (WR52_27275) named HN001-Δ*fla* and HN001-Δ*plcR*, respectively, were constructed in this study. The *fla* and *plcR* genes were deleted through homologous recombination using the plasmid pKMBKI, a temperature-sensitive shuttle vector that can replicate in both *E. coli* and *B. cereus* [23]. The upstream and downstream homologous fragments of *fla* and *plcR* were ligated with the spectinomycin resistance gene, and the resulting fragments were inserted into pKMBKI. The verified plasmids were introduced into HN001 via electroporation. Transformants were selected at 30°C on LB agar plates containing chloramphenicol and spectinomycin. A single colony was then transferred to liquid medium without antibiotics and grown at 42°C for four generations. The colonies were then plated onto LB agar plates containing only spectinomycin, and subsequently incubated on chloramphenicol LB agar plates to identify those resistant to spectinomycin but not chloramphenicol. The *fla* and *plcR* deletion mutants were validated via PCR and sequencing analyses. All bacterial strains, plasmids, and primers used in this work are shown in Tables S1 and S2.

### Mice and cells

All animals used in this study were housed at the animal center of the Academy of Military Medical Sciences (AMMS). Animals were cared for in accordance with the principles of laboratory animal care approved in China. The protocol was approved by the Institutional Animal Care and Use Committee of AMMS. WT C57BL/6 mice were obtained from Beijing Vital River Laboratory Animal Technology. The Gb3-deficient mice (*A4galt*^−/-^) used in this study were generated in a previous study [24].

The human cerebral microvascular endothelial cell line hCMEC/D3 was obtained under license from INSERM, France. Gb3-deficient (*A4GALT*^−/-^) hCMEC/D3 cells were generated via CRISPR-Cas9 gene editing as previously described [25], to eliminate exon 3 of the human A4GALT locus (sgRNA, GTCCGGGGCGCCCCAAGGCAG) (Figure S1). The *A4GALT*^−/-^ cell line was identified via flow cytometry using anti-CD77 antibody [38-13] (ab 19795, Abcam) (Figure S1). hCMEC/D3 cells were cultured as described previously [26]. CaCo-2 cells were obtained from China Infrastructure of Cell Line Resource and cultured in minimum essential medium (Gibco) supplemented with 20% fetal bovine serum (Gibco) and non-essential amino acids (Gibco). Cells were incubated in a humidified incubator containing 5% CO_2_ at 37°C.

### Mouse infection

*B. cereus* infection in adult C57BL/6 mice was performed as previously described with some modifications [27]. The overnight cultures of *B. cereus* were subcultured (1:100) in fresh BHI medium, and mid-log phase bacteria were washed three times with PBS. Mice (6–8 weeks old) were intraperitoneally injected with 5 × 10^6^ colony-forming units (CFUs) of *B. cereus*.

### CFU assay

The bacterial loads in livers and spleens after infection were determined by plating serial dilutions of tissue homogenates onto LB agar plates. Briefly, the organs were washed with sterile PBS, weighed, and homogenized in sterile PBS, and 100 μl of serial dilutions were plated and incubated at 37°C for 18 h. The bacterial load was represented as the number of CFUs per 0.05 g of tissue.

### Cytokine analysis

Cytokine levels of mouse serum were determined using a ProcartaPlex™ Multiplex Immunoassay (EPX200-26090-901, Invitrogen) according to the manufacturer’s instructions.

### Histopathological assessment

Livers from infected mice were removed and fixed in neutral buffered 4% formalin. Sections were stained with hematoxylin and eosin, and the histopathology of infected organs was analyzed using an Olympus BX53 microscope.

### Purification and analysis of the galabiose-binding proteins of *B. cereus*

Pigeon ovomucoid (POVO) containing the terminal Galα1-4Gal residues and *B. cereus* cell wall extract were purified as described previously [11]. Briefly, POVO was coupled to Dynabeads® M-280 Tosylactivated (14023, Invitrogen) in accordance with the manufacturer’s instructions. The *B. cereus* cell wall extract was incubated with POVO-coupled Dynabeads at 8°C for 2 h with gentle mixing. The Dynabeads were washed three times with PBS and then boiled in 60 μl of protein loading buffer for 10 min to separate the galabiose-binding proteins from the Dynabeads. Proteins in the supernatant were analyzed via SDS-PAGE, the gel was silver-stained and the protein bands were processed for MALDI-TOF-MS analysis.

### Adhesion assay

Mid-log phase *B. cereus* specimens were washed with PBS and then resuspended in cell culture medium without antibiotics. Cells grown in 24-well plates were infected with the bacterial suspension at an MOI of 10:1. After 1 h, cells were washed three times with PBS to remove the non-attached bacteria and then lysed with 0.1% saponin on ice for 15 min. The number of cell-associated *B. cereus* specimens was determined by plating serial dilutions of the lysate on LB agar plates. The percentage of cell-associated *B. cereus* specimens was calculated as (recovered CFU/inoculated CFU) × 100%.

### Flow cytometry

Cells were incubated with Alexa Fluor 647-labeled flagellin for 30 min at room temperature, followed three times washes in FACS buffer. Flow cytometric data acquisition was performed on a BD FACSVerse™ flow cytometer (BD Biosciences). Data were analyzed using FlowJo software.

### Preparation of recombinant proteins

The gene fragments of flagellin (WR52_08290), triosephosphate isomerase (WR52_26170), and superoxide dismutase (WR52_27745) were amplified by PCR from the genome of *B. cereus* HN001 (GenBank No. CP011155.1). The primers used to amplify these fragments are listed in Table S2. These three fragments were separately inserted into the pET28a vector (Novagen). The resulting plasmids were transformed into *E. coli* strain BL21 (DE3), and transformation was confirmed by DNA sequencing. The expression of recombinant proteins was incubation with 1 mM isopropyl-β-D-thiogalactopyranoside at 37°C for 4 h. The proteins were purified using a Ni-chelating chromatography column (GE Healthcare) according to the manufacturer’s instructions.

### Electron microscopy

To visualize the flagella of *B. cereus*, we performed transmission electron microscopy. Specifically, 2.5% glutaraldehyde-fixed bacterial suspensions were placed on grids and negative stained with a 2% solution of phosphotungstic acid. The samples were examined using a Hitachi H1650 transmission electron microscope. For scanning electron microscopy, *B. cereus*-infected cells were washed three times with PBS to remove the non-attached bacteria, and then sample preparation was performed as described previously [28]. The samples were examined using a Hitachi S3400 N scanning electron microscope.

### Confocal microscopy

For immunofluorescence microscopy, infected CaCo-2 cells were fixed with 4% paraformaldehyde in PBS, and flagellin was stained with rabbit anti-flagellin and Alexa Fluor 488-conjugated goat anti-rabbit IgG (Thermo Fisher), The samples were imaged using a confocal laser-scanning microscope (Olympus FV1000).

### Immunoblot analysis

Natural flagellins were extracted from the surface of *B. cereus* HN001 as described previously [29]. To detect natural flagellins, 300 μl of the supernatant of the extract were mixed with 100 μl of sample loading buffer. A primary antibody against flagellin (1:1,000 dilution) and goat anti-rabbit (IgG) secondary antibody (1:5,000 dilution, Thermo Fisher Scientific) were used.

### Statistical analysis

GraphPad Prism 7 software was used for statistical analyses. Data are presented as the mean ± SD. The log-rank test was used to compare the mouse survival curves. Comparisons of two groups were performed using an unpaired two-tailed Student’s *t* tests, and comparisons of more than two groups were performed via one or two-way ANOVA. *P* < 0.05 was considered statistically significant.

## Results

### Mice that cannot produce the glycosphingolipid Gb3 experience reduced lethality from *B. cereus* infection

**Figure 1.**
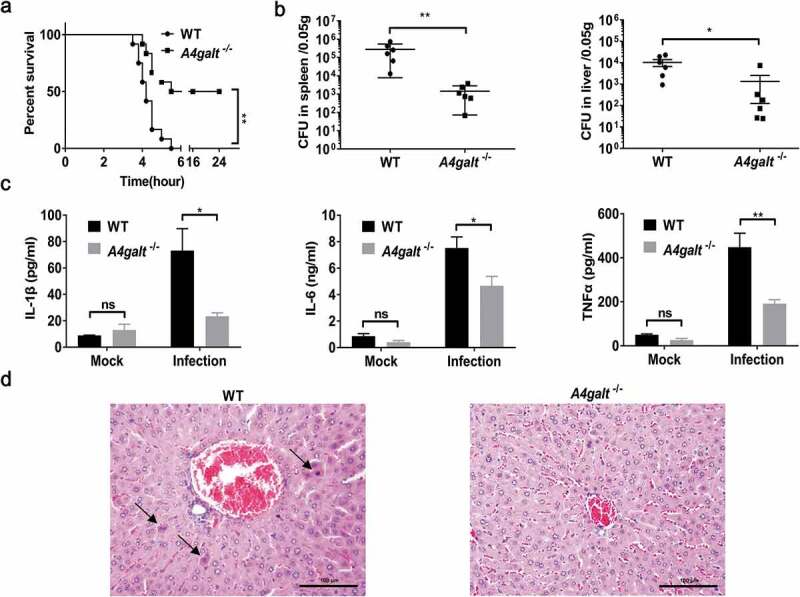
Mice lacking the glycosphingolipid Gb3 experience reduced lethality from *B. cereus* infection. WT and Gb3-deficient (*A4galt*^−/-^) mice were intraperitoneally injected with *B. cereus* strain HN001 (5 × 10^6^ CFU/mouse). (a) Survival of mice infected with *B. cereus* (log-rank test, n = 12). The bacterial loads in the spleens and livers (b), and cytokine levels in serum (c) at 4 h post-infection were determined by colony plate count (two-tailed, unpaired *t* tests, n = 6) and ProcartaPlex™ multiplex immunoassay (Two-way ANOVA, n = 4). Mock infection (Mock) as a control. (d) Histopathology analysis of liver tissue from WT mice and *A4galt*^−/-^ mice at 4 h post-infection, black arrow showed the apoptotic cells in liver. The data are shown as the mean ± SD (*P < 0.05 and **P < 0.01).

Several studies have employed *B. cereus* infection models and monitored the survival of mice [27,30]. We infected WT and Gb3-deficient (*A4galt*^−/-^) C57BL/6 mice with 5 × 10^6^ CFUs of *B. cereus* HN001 by intraperitoneal (i.p.) injection. At 6 h post-infection, all of the WT mice died (n = 12), but 6 of 12 (50%) of the *A4galt*^−/-^ mice survived (Figure 1(a)). Analyses of the bacterial loads in the spleens and livers showed that the CFU in WT mice was significantly higher than that in the *A4galt*^−/-^ mice (Figure 1(b)). Furthermore, WT mice infected with *B. cereus* HN001produced significantly more IL-1β, IL-6, and TNFα in serum compared with *A4galt*^−/-^ mice (Figure 1(c)), indicating the inflammation in WT mice was more severe than that in *A4galt*^−/-^ mice. Histologically, the livers of infected WT mice showed more apoptotic cells than *A4galt*^−/-^ mice (Figure 1(d)). These results clearly suggest that Gb3 is somehow involved in the *B. cereus* infection process in mice.

### *Surface components of* B. cereus *involved in the interaction with Gb3*

**Figure 2.**
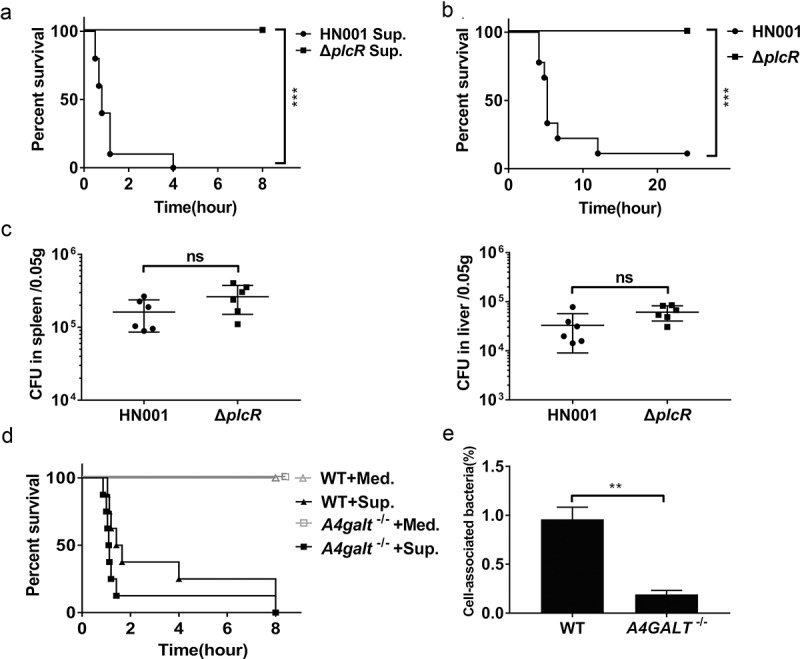
*B. cereus* interacts with Gb3 as it adheres to the surface of host cells. (a) Survival of WT mice after intraperitoneal injection with *B. cereus* HN001 culture supernatant (Sup.) or HN001-Δ*plcR* culture supernatant (Δ*plcR* Sup.) (log-rank test, n = 10). (b) Survival of WT mice after intraperitoneal infection with 5 × 10^6^ CFU *B. cereus* HN001 or HN001-Δ*plcR* (Δ*plcR*) (log-rank test, n = 10). (c) WT mice were intraperitoneal injected with 5 × 10^6^ CFU *B. cereus* HN001 or HN001-Δ*plcR*, the bacterial loads in spleens and livers at 2 h post-infection was assessed (two-tailed, unpaired *t*-tests, n = 6). (d) Survival of WT and *A4galt*^−/-^ mice after intraperitoneal injection with 1 ml of *B. cereus* BHI medium (Med.) or supernatant prepared from BHI-grown *B. cereus* cultures (Sup.) (log-rank test, n = 8). (e) WT and Gb3-deficient hCMEC/D3 cells (*A4GALT*^−/-^) were infected with *B. cereus* (MOI = 10:1), the adherence rate was calculated by colony plate counting. The data are shown as the mean ± SD (**P < 0.01 and ***P < 0.001).

It is known that *B. cereus* secretes many virulence factors such as enterotoxins, hemolysins, phospholipases, beta-lactamases, proteases, collagenases, and emetic toxin (cereulide) [31]. Whole-genome sequencing of the *B. cereus* strain HN001 showed that its genome harbors loci for production of hemolysin BL (Hbl), non-hemolysin enterotoxin (Nhe), cytotoxin K (CytK), enterotoxin FM (EntFM), and sphingomyelinase (SMase), but it does not have any obvious homologous genes of the cereulide synthetase gene cluster. Previous studies indicated that secreted Hbl and SMase promote the death of *B. cereus* infected mice [27,30], and thus we injected the supernatant of *B. cereus* HN001 cultures into the peritoneal cavities of WT mice, which caused death within 4 h (Figure 2(a)).

PlcR has been characterized as one of *B. cereus*’s major virulence transcription factors: it has been shown to positively regulate the transcription of genes for 22 secreted proteins (*e.g*., toxins, phospholipases, proteases, peptides) [32]. We therefore knocked out *plcR* from the HN001 strain and found that the injection of WT mice with the supernatant of HN001-Δ*plcR* cultures did not kill any mice (Figure 2(a)). These results indicate that some of the PlcR-regulated secreted virulence factors are responsible for the death of the injected mice. When we infected the WT mice with *B. cereus* HN001 and HN001-Δ*plcR* cells, 9 of the 10 mice infected with HN001 died, whereas no died upon infection with HN001-Δ*plcR* (Figure 2(b)). Notably, we detected no difference in the bacterial loads in the spleens or livers of mice infected with HN001 or HN001-Δ*plcR* (Figure 2(c)). These results imply that the bacterial load *per se* is not the direct cause of death in *B. cereus* infected mice.

As with WT mice, *A4galt*^−/-^ mice died within 8 h of injection with the supernatant of HN001 cultures (Figure 2(d)). However, recalling our aforementioned finding that the *A4galt*^−/-^ mice experienced reduced lethality upon *B. cereus* infection compared to WT mice (Figure 1(a)), these results establish that Gb3 is not apparently involved in the secreted-virulence-factor-mediated death of mice. We therefore speculated that some interaction between the surface of the pathogenic bacterium and the host cell, rather than any secreted virulence factors, can account for the observed reduction in the lethality of *B. cereus* infection for WT vs. *A4galt*^−/-^ mice. Pursuing this hypothesis, we conducted assays using hCMEC/D3 cells, which have been revealed to accumulate Gb3 on their surface [24]. Specifically, we evaluated the adhesion ability of *B. cereus* to WT hCMEC/D3 cells and to Gb3-deficient hCMEC/D3 cells (generated via CRISPR-Cas9, *A4GALT*^−/-^). The adhesion assay indicated that the number of cell-associated bacteria was significantly lower in *A4GALT*^−/-^ cells than in WT cells (Figure 2(e)). Collectively, these results indicate that surface components of *B. cereus* engage in interactions with the Gb3 glycosphingolipid on the surface of host cells and suggest that this interaction somehow promotes the lethality of *B. cereus* infection.

### The *B. cereus* flagellin protein interacts with Gb3 on the surface of host cells

**Figure 3.**
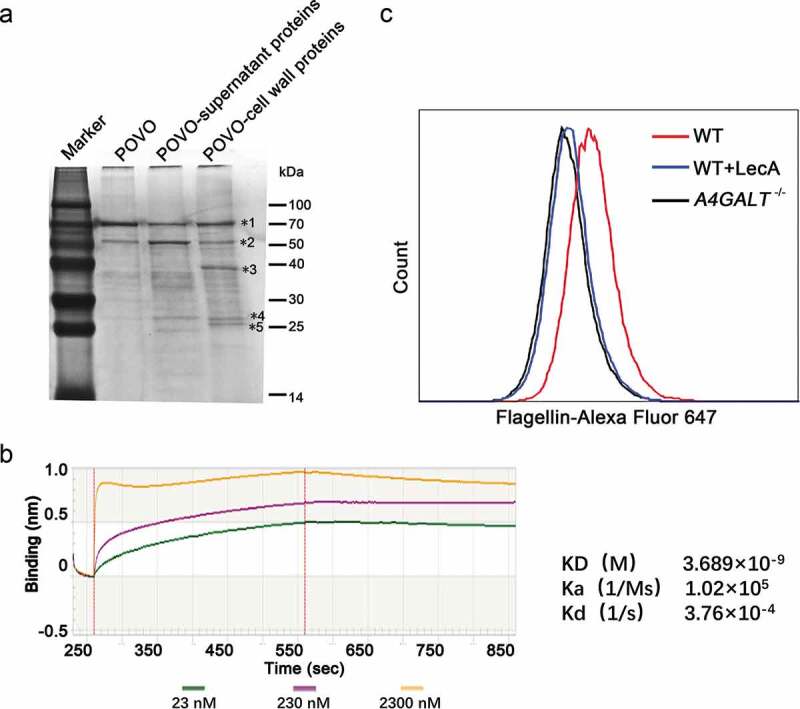
Identification of the interaction between flagellin and Gb3. (a) Silver staining of the *B. cereus* cell wall proteins binding to pigeon ovomucoid (POVO). The control groups using pigeon ovomucoid-coupled Dynabeads with *B. cereus* culture supernatant proteins or without *B. cereus* proteins. The major components of the main bands (*1–*5) were analyzed via MALDI-TOF-MS. (b) The affinity constant of flagellin-ovomucoid was measured via BLI (bio-layer interferometry) technology using a BLItz system. (c) WT hCMEC/D3 cells, LecA pre-treated WT hCMEC/D3 cells, and Gb3-deficient hCMEC/D3 cells (*A4GALT*^−/-^) were incubated with Alexa Fluor 647-labeled flagellin, and interaction(s) between flagellin and hCMEC/D3 cells were analyzed via flow cytometry. Data was analyzed using FlowJo software.

Kouki et al. identified the Gb3 binding protein SadP, a *S. suis* cell wall protein, with an experimental strategy based on a matrix containing oligosaccharides containing the terminal Galα1-4Gal sequence (POVO) and LC-ESI mass spectrometry [11]. We adopted a similar approach using POVO to identify Gb3-interacting proteins, as the Galα1-4Gal (galabiose) moiety of Gb3 is the binding site by bacterial virulence factors. Briefly, we used Dynabeads conjugated to POVO to capture components from *B. cereus* cell wall extracts. After SDS-PAGE and silver staining, the major components of the main bands were analyzed via mass spectrometry (Figure 3(a)). The 70 and 50 kDa bands (Figure 3(a(*1,*2))) were identified to primarily contain BSA (69.3 kDa) and ovomucoid (50 kDa) respectively, two protein components of the Dynabeads system (i.e., not originating from the *B. cereus* cell wall extracts). The 40, 26 and 25 kDa bands (Figure 3(a(*3–*5))) were found to contain the bacterial proteins flagellin (39.1 kDa), triosephosphate isomerase (26.5 kDa), and superoxide dismutase (24.0 kDa), respectively.

We next expressed recombinant flagellin, triosephosphate isomerase, and superoxide dismutase and purified them using Ni-chelating chromatography. To verify the interactions of these three proteins with Galα1-4Gal-containing POVO, we measured affinity constants for the three proteins-ovomucoid binding interactions using biotinylated POVO. The affinity constant of flagellin-ovomucoid was 3.689 × 10^–9^ M (Figure 3(b)), which confirmed the interaction between flagellin and ovomucoid. The affinity constants of triosephosphate isomerase-ovomucoid and superoxide dismutase-ovomucoid were low (data not shown), and thus we subsequently focused on flagellin.

To investigate whether flagellin binds to Gb3 on the cell surface, Alexa Fluor 647-labeled flagellin was incubated with WT and *A4GALT*^−/-^ hCMEC/D3 cells. WT hCMEC/D3 cells were pre-treated with a high concentration of LecA – a protein known to bind Gb3 with strong affinity [9] – to preclude flagellin binding with Gb3. We then used flow cytometry targeting Alexa Fluor-647 labeled flagellin to investigate the interaction between flagellin and Gb3 on the surface. The *A4GALT*^−/-^ hCMEC/D3 cells and LecA pre-treated WT hCMEC/D3 cells showed much lower fluorescence intensity compared with the untreated WT hCMEC/D3 cells (Figure 3(c)), findings showing that Gb3 and flagellin do indeed interact directly.

### The interaction of flagellin with Gb3 contributes to the adherence of *B. cereus* to host cells

**Figure 4.**
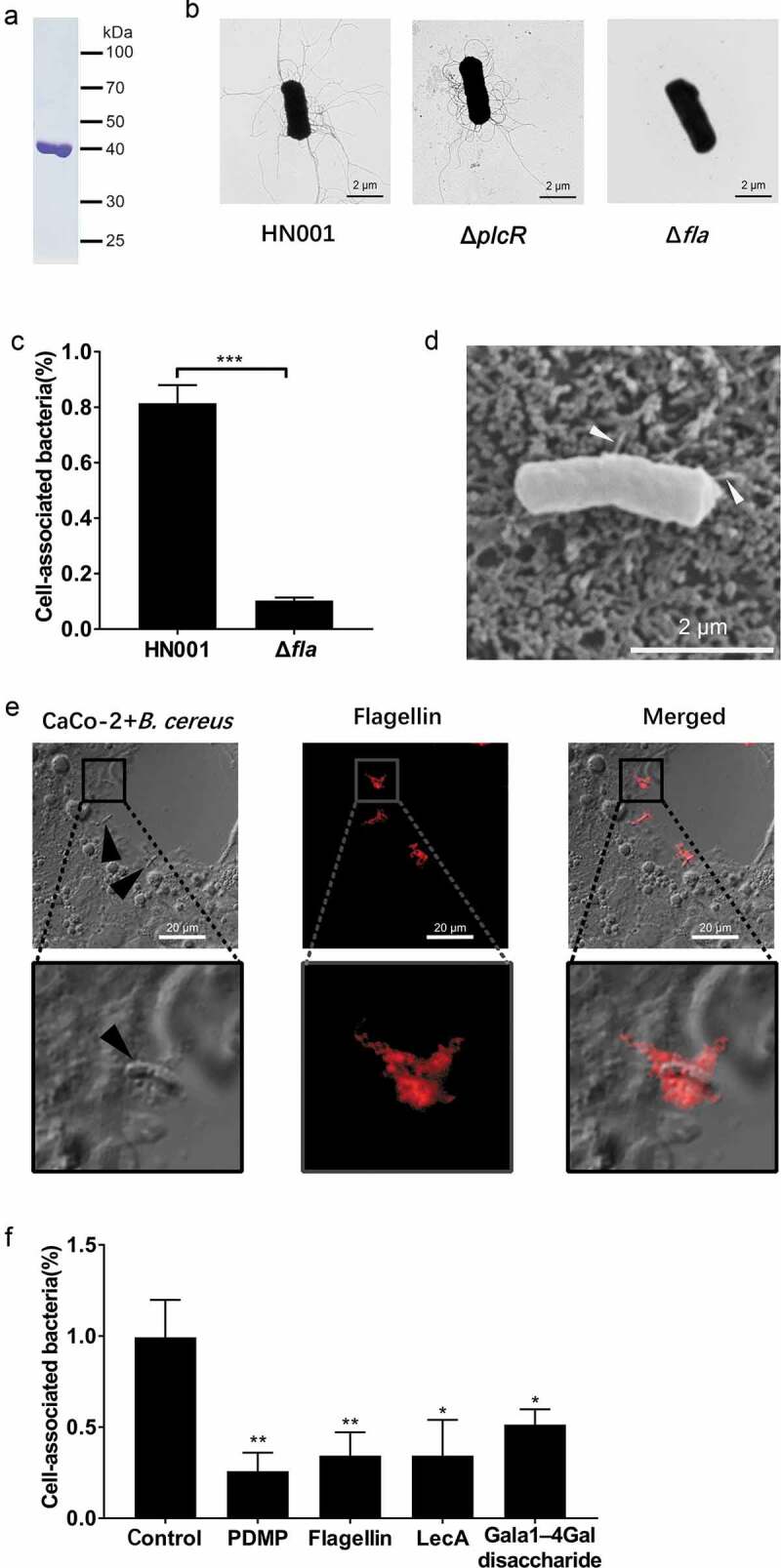
The interaction of flagellin with Gb3 contributes to the adherence of *B. cereus* to the host. (a) Flagellin extracted from the surface of *B. cereus* HN001 cells. (b) Negative‐stain transmission electron images of HN001 and the flagellin knockout strain HN001-Δ*fla* (Δ*fla*). (c) CaCo-2 cells were infected with HN001 or HN001-Δ*fla* (MOI = 10:1), the adherence rate was calculated by colony plate counting. (d, e) Scanning electron microscopy and laser confocal microscopy of the adherence of *B. cereus* to the host cell surface (white arrowheads, flagella filaments; black arrowheads, *B. cereus* cells; red, flagellin). (f) Unmodified CaCo-2 cells or CaCo-2 cells pre-treated with PDMP, flagellin, LecA, or Galα-4Gal infected with HN001 (MOI = 10:1); the adherence rate was calculated by colony plate counting. The data are shown as the mean ± SD (*P < 0.05, **P < 0.01 and ***P < 0.001).

The flagellin protein is the major structural component of bacterial flagellar filaments, and flagella have been reported to function as adhesins [19]. The number of distinct flagellin subunit types comprising flagella filaments of *B. cereus* is known to vary by strain, with *B. cereus* ATCC 14579 producing three flagellin subunits [29,33]. According to the whole-genome sequence analysis of *B. cereus* HN001, there are two flagellin genes, namely WR52_08285 and WR52_08290, are present in the genome of the HN001 strain. We extracted the natural flagellins from the surface of *B. cereus* HN001 and observed a single 39 kDa band product (Figure 4(a)). This band was identified as Bacillus flagellin (encoded by the WR52_08290 locus) by mass spectrometry, suggesting that the HN001 strain expresses only one 39 kDa flagellin. To investigate the role of flagellin in the adhesion of *B. cereus*, we constructed a *B. cereus* flagellin (encoded by the WR52_08290 locus) knockout strain (HN001-Δ*fla*). *B. cereus* HN001 and HN001-Δ*plcR* cells had many flagellar filaments, whereas the HN001-Δ*fla* cells had none (Figure 4(b)).

CaCo-2 cells, a colon cancer cell line (colorectal adenocarcinoma epithelial cells), have been shown to express Gb3 on its surface [34]. We conducted an adhesion assay using CaCo-2 cells and found that the ability of HN001-Δ*fla* cells to adhere to CaCo-2 cells was significantly reduced compared to HN001 cells (Figure 4(c)). Scanning electron microscopy and laser confocal microscope were used to observe *B. cereus* adhering to the CaCo-2 cell surface (Figure 4(d,e)). These results imply that flagellin contributes to the adherence of *B. cereus* to the host.

To investigate whether *B. cereus* adheres to the cell surface based on interaction between flagellin and Gb3, we used a combination of two methods to prepare these cells for experiments: pre-treatment of CaCo-2 cells with D, L-*erythro*-1-phenyl-2-decanoylamino-3-morpholino-1-propanol•HCl PDMP (27 μM, 3 days) [35,36] to block the biosynthesis of Gb3, or pre-treatment with a high concentration of LecA (3 μg/ml) or purified flagellin (3 μg/ml) to preclude *B. cereus* from binding to Gb3. We then conducted an adhesion assay using colony plate counting and found that pre-treated CaCo-2 cells associated with significantly fewer *B. cereus* cells than did unmodified CaCo-2 cells (Figure 4(f)). We also conducted experiments in which the disaccharide Galα-4Gal (a competing ligand to Gb3, 0.5 mM) was added to saturate potential binding with flagellin, and again, the pre-treated cells associated with significantly fewer *B. cereus* cells (Figure 4(f)). These results together indicate that *B. cereus* adheres to host cells based at least in part on an interaction between flagellin and the Gb3 glycosphingolipid on the surface of host cells.

### Flagellin knockout decreases the lethality of *B. cereus*in a murine infection model

**Figure 5.**
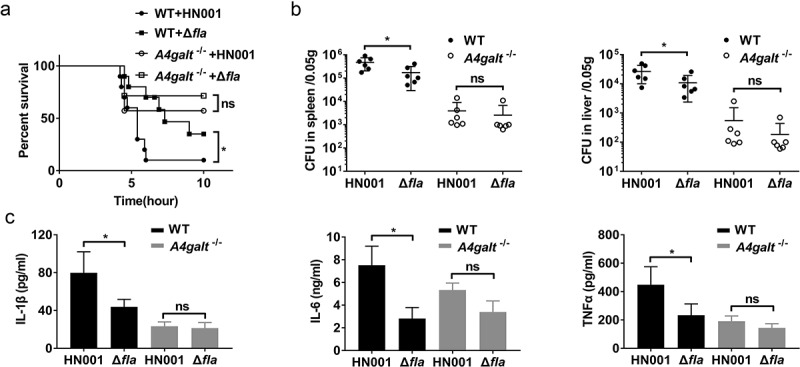
Flagellin knockout decreases the lethality of *B. cereus* in a murine infection model. WT and *A4galt*^−/-^ mice were intraperitoneally injected with the *B. cereus* strain HN001 or HN001-Δ*fla* (Δ*fla*). (a) Survival of mice infected with *B. cereus* (5 × 10^6^ CFU/mouse, log-rank test, n = 10). (b) Detection of the bacterial loads in the spleens and livers at 4 h post-infection (two-tailed, unpaired *t-*tests, n = 6). (c) Determination of cytokine levels in serum at 4 h post-infection (two-tailed, unpaired *t*-tests, n = 4). The data are shown as the mean ± SD (*P < 0.05).

Adhesion of bacteria to host cells is a key event during infection, thus, to investigate the role of flagellin in *B. cereus* infection in mice, we infected WT and *A4galt*^−/-^ mice with HN001 and HN001-Δ*fla*. The HN001-Δ*fla* infected WT mice survived longer and had lower mortality than HN001 infected WT mice (Figure 5(a)), illustrating that flagellin directly participates in *B. cereus* infection in mice. We also noted that the bacterial loads in the spleens and livers of HN001-Δ*fla* infected WT mice were significantly lower than those in HN001 infected WT mice (Figure 5(b)). Further, WT mice infected with HN001-Δ*fla* produced significantly reduced levels of IL-1β, IL-6, and TNFα in serum compared with WT mice infected with HN001 (Figure 5(c)). However, the HN001 and HN001-Δ*fla* infected *A4galt*^−/-^ mice had no significant difference in mortality, bacterial loads in the spleens and livers, and the levels of IL-1β, IL-6, and TNFα in serum (Figure 5(a–c)). These results indicate that flagellin might contribute to the *B. cereus* infection process of mice in the presence of Gb3.

### An anti-flagella antibody confers protection against *B. cereus* infection

**Figure 6.**
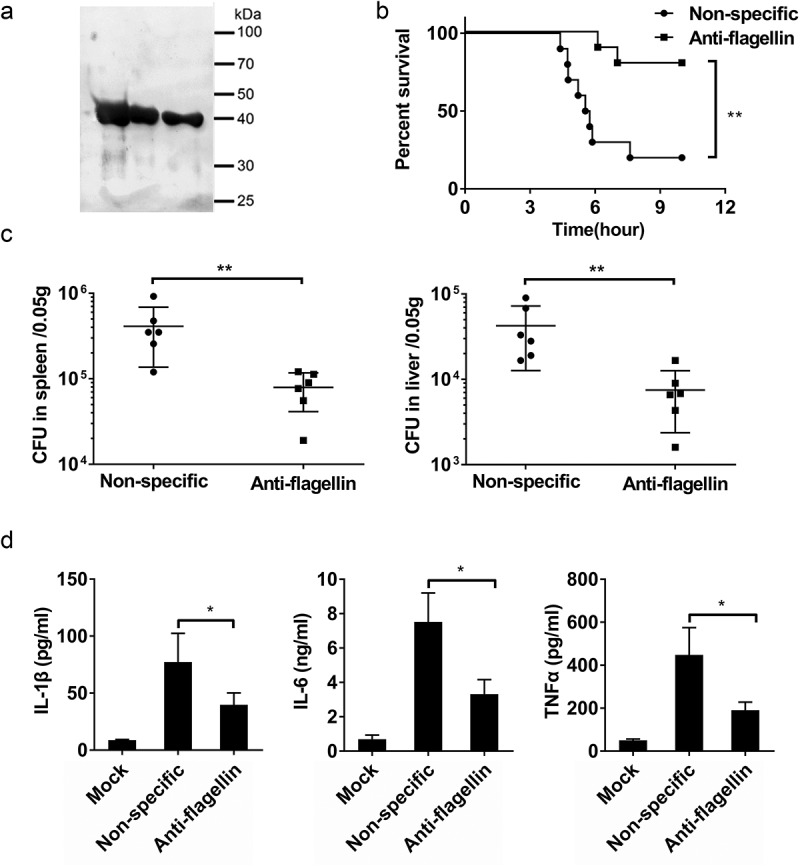
An anti-flagellin antibody protect mice against *B. cereus* infection. (a) To generate a polyclonal antibody against *B. cereus* flagellin (anti-flagellin), rabbits were immunized with flagellin of *B. cereus*; the anti-flagellin polyclonal antibody were then purified from antisera. The anti-flagellin polyclonal antibody were used for immunoblot analysis of natural flagellin. (b–d) WT mice were intraperitoneally injected with nonspecific antibody (nonspecific) or anti-flagellin antibody before infection with *B. cereus* HN001 (5 × 10^6^ CFU/mouse). (b) Survival of mice infected with *B. cereus* HN001 (log-rank test, n = 10). (c) Detection of the bacterial loads in the spleens and livers at 4 h post-infection (two-tailed, unpaired *t-*tests, n = 6). (d) Determination of cytokine levels in serum at 4 h post-infection (two-tailed, unpaired *t*-tests, n = 4). Mock infection (Mock) as a control. The data are shown as the mean ± SD (*P < 0.05 and **P < 0.01).

Previous studies reported that anti-flagellin antibodies can effectively reduce mouse mortality and morbidity attributable to bacterial infections[37,38]. Barnea et al. found that treatment with an anti-flagellin type-a antibody limits the invasiveness of *P. aeruginosa* in a murine *P. aeruginosa* infected burn model [37]. To evaluate whether an anti-flagellin antibody can affect the ability of *B. cereus* to infect host cells, we immunized rabbits with *B. cereus* flagellin and purified anti-flagellin polyclonal antibody from antisera (Figure 6(a)). WT C57BL/6 mice were then treated with rabbit anti-flagellin or nonspecific antibody and then were infected with HN001. The mice given the anti-flagellin antibody mice displayed lower mortality (Figure 6(b)), lower bacterial loads in the spleens and livers (Figure 6(c)), and reduced IL-1β, IL-6, and TNFα levels in serum compared to mice given the nonspecific antibody (Figure 6(d)). These results indicate that anti-flagellin antibody can protect mice against *B. cereus* infection.

## Discussion

Previous studies reported that several bacteria and viruses use Gb3 as a binding site on the host cell surface. In this study, we confirmed that the presence of Gb3 exacerbated *B. cereus* infection in mice. Moreover, we demonstrated that *B. cereus* adhered to host cells via an interaction between flagellin and Gb3. The deficiency of flagellin or treatment with an anti-flagellin antibody reduced the adhesion of bacteria to host cells and promoted mouse survival following infection. Taken together, these results suggested that the interaction between flagellin and Gb3 was essential for *B. cereus* to adhere to host cells, and the interaction contributed to acute *B. cereus* infection.

The pathogenicity of *B. cereus* in intestinal or non-intestinal infections is intimately associated with the ability of the bacterium to produce toxins [31]. Previous studies reported that Hbl induced overt activation of the NLRP3 inflammasome and promoted the rapid death of mice [27]. Our studies also revealed that secreted virulence factors were responsible for the death of the injected mice; however, the secreted virulence factors could not account for the observed reduction in the lethality of *B. cereus* infection in *A4galt*^−/-^ mice. Previous studies confirmed that factor H-binding protein, a ligand of Gb3, contributed to the adhesion of *S. suis* to hCMEC/D3 cells and promoted the development of meningitis and subsequent mortality in mice [24]. In this study, our results suggested that Gb3 promoted bacterial adhesion to host cells and contributed to acute infection. The attachment of *B. cereus* to the epithelial surface or host mucus layer results in the production of several virulence factors [39–41]. Therefore, we speculated that some bacterial cell surface components interact with Gb3, and the interactions may be involved in the adhesion and colonization of *B. cereus*, leading to increased pathogenicity.

The adhesion of *B. cereus* to host cells contributes to its virulence [42]. Gb3, a receptor expressed on many cell surfaces, has been reported to participate in the adhesion of bacteria to host cells. Ferrando et al. confirmed that SadP, a ligand of Gb3, contributed to *S. suis* adhesion to human epithelial cells [13]. In various bacterial species, such as *E. coli, P. aeruginosa*, and *Salmonella enterica* serovar Typhimurium, flagella and flagellin have been reported to function as adhesins [19]; however, the receptors have not been determined in most of these species. Without a flagellar basal body protein encoded by flhA, *Bacillus thuringiensis* cannot assemble flagellar filaments, resulted impaired adhesion [15,43]. Our results revealed that *B. cereus* adhered to CaCo-2 cells via an interaction between flagellin and Gb3; moreover, this adhesion might contribute to the colonization of *B. cereus* infection in mice.

Passive antibody therapy is currently used to treat and prevent many infectious diseases. Regarding bacteria, the targets for antibodies include secreted virulence factors and bacterial surface molecules [44]. One of the modes of action of antibacterial antibodies is the inhibition of adhesion. In uropathogenic *E. coli*, FimH is a lectin-like protein located at the tip of type 1 fimbriae. Kisiela et al. demonstrated that anti-FimH antibody could inhibit bacterial adhesion and protect mice against urinary tract infection [45,46]. Our studies revealed that anti-flagellin polyclonal antibody rescued mice from *B. cereus* infection, although an antibody might not inhibit bacterial adhesion. Previous studies found that an anti-flagellin type-a monoclonal antibody effectively reduced mortality and morbidity in a murine mouse burn wound sepsis model [37]. These findings suggest that flagellin is an appropriate antibody target because the flagellin proteins have been described in many pathogens.

In conclusion, we validated that the receptor Gb3 contributed to acute *B. cereus* infection in mice. We also demonstrated that the interaction between flagellin and Gb3 promoted the adhesion of *B. cereus* to host cells *in vitro*. Our results further indicated that passive antibody therapy using anti-flagellin antibody represented an alternative therapeutic option for *B. cereus* infection.

## Supplementary Material

Supplemental Material
