# Supplementary material for: The interaction between flagellin and the glycosphingolipid Gb3 on host cells contributes to Bacillus cereus acute infection
Source: Virulence. 2020 Jun 7;11(1):769–80. doi: 10.1080/21505594.2020.1773077 (PMC7567440; doi:10.1080/21505594.2020.1773077)
Supplement: Supplemental Material [file KVIR_A_1773077_SM2623.zip › Figure S1 caption.docx]

**Figure S1. Gb3-deficient hCMEC/D3 cells (*A4GALT*^-/-^) were generated via CRISPR-Cas9.** (A) Sequencing chromatograms of the *A4GALT* gene in wild-type and *A4GALT*^-/-^ hCMEC/D3 cells. (B) The nucleotide and amino acid sequences of the *A4GALT* transcript in *A4GALT*^-/-^ cells are shown. Red indicates the mutated amino acids achieved by deletion “GC”. (C) Wild-type and *A4GALT*^-/-^ hCMEC/D3 cells were stained with anti-CD77 antibody. A control using isotype-matched antibody was performed in parallel. Flow cytometric data acquisition was performed on BD FACSVerse™ flow cytometer (BD Biosciences). Data was analyzed using FlowJo software.
