# Supplementary material for: The interaction between flagellin and the glycosphingolipid Gb3 on host cells contributes to Bacillus cereus acute infection
Source: Virulence. 2020 Jun 7;11(1):769–80. doi: 10.1080/21505594.2020.1773077 (PMC7567440; doi:10.1080/21505594.2020.1773077)
Supplement: Supplemental Material [file KVIR_A_1773077_SM2623.zip › Table S1.docx]

**Table S1. Bacterial strains and plasmids used in this study.**

| Plasmids and strains | Description | Source/Reference |
| --- | --- | --- |
| Plasmids |  |  |
| pKMBKI | Shuttle vector, Amp^r^ in *E. coli* and Cm^r^ in *B. cereus* | [1] |
| pSET4s | Shuttle vector, Spc^r^ both in *E. coli* and *B. cereus* | [2] |
| pET28a | Vector for overexpressing His-tagged proteins, Kan^r^ | Novagen |
| LentiCRISPR v2 | Plasmid for CRISPR/Cas9 genome editing | [3] |
| *B. cereus* strains |  |  |
| HN001 | Wild-type *B. cereus* | This lab |
| HN001-Δ*fla* | HN001 with *fla* gene knocked out and Spc^r^ gene instead | This study |
| HN001-Δ*PlcR* | HN001 with *plcR* gene knocked out and Spc^r^ gene instead | This study |
| *E. coli* strains |  |  |
| DH5α | Cloning strain | TransGen, China |
| BL21(DE3) | Strain used to express recombinant proteins | TransGen, China |
| SCS110 | *dam*–/*dcm*– strain used to produce unmethylated plasmid | TransGen, China |

**References:**

1. Wang, T., et al., *Construction of a high-efficiency cloning system using the Golden Gate method and I-SceI endonuclease for targeted gene replacement in Bacillus anthracis.* J Biotechnol, 2018. 271: p. 8-16.

2. Takamatsu, D., M. Osaki, and T. Sekizaki, *Thermosensitive suicide vectors for gene replacement in Streptococcus suis.* Plasmid, 2001. 46(2): p. 140-8.

3. Ran, F.A., et al., *Genome engineering using the CRISPR-Cas9 system.* Nature protocols, 2013. 8(11): p. 2281-2308.
