# Supplementary material for: The interaction between flagellin and the glycosphingolipid Gb3 on host cells contributes to Bacillus cereus acute infection
Source: Virulence. 2020 Jun 7;11(1):769–80. doi: 10.1080/21505594.2020.1773077 (PMC7567440; doi:10.1080/21505594.2020.1773077)
Supplement: Supplemental Material [file KVIR_A_1773077_SM2623.zip › Table S2.docx]

**Table S2. Primers used in this study.**

| Name | Sequence (5′→3′) |
| --- | --- |
| spc_F | CCGGAATTCGAGCTCCTCGAGGTGTTCGTGAATACATGTTATA |
| spc_R | CCGGGATCCGGTACCGCTAGCGTTTTCTAAAATCTGATTACCA |
| fla_up_F | CCGGAATTCGAGCTCCATATGTACGACTTACACGAT |
| fla_up_R | GAGCTCCTCGAGGTTAAGAAACCCCCATTTTTTTA |
| fla_dn_F | GGTACCGCTAGCGCCATTTGAGTTAGTTTCCTG |
| fla_dn_R | CGCGGATCCATCATACTAGTTGCAACTTCAGT |
| plcR_up_F | TTCGAGCTCAGTTCCTTGCTCACTTGTAG |
| plcR_up_R | CCGCTCGAGACTTACTCACCATCCCATTAG |
| plcR_dn_F | CGGGCTAGCAAAACAACCGTCTTACTTAGG |
| plcR_dn_R | CGCGGATCCTATCGTGCTAAAGGAAATGG |
| pKMBKI _F | ATGTGCTGCAAGGCGATTA |
| pKMBKI_R | CCCAGGCTTTACACTTTATG |
| fla_seq_F | TTTCCCAGTTCCTTGCTCACTTGTAG |
| fla_seq_R | ACCAAGTATCGGAATTGCGATGTACC |
| plcR_seq_F | TTTCCCAGTTCCTTGCTCACTTGTAG |
| plcR_seq_R | ACCAAGTATCGGAATTGCGATGTACC |
| fla_F | CCATATGAGAATTAATACAAACATTAACAG |
| fla_R | CCTCGAGTTATTGTAATAATTTAGAAACC |
| tpiA_F | CCATATGATGCGTAAACCAATTATCGCAGG |
| tpiA_R | TCTCGAGTCATTTTACCGCCCCCAGA |
| sod_F | AGGATCCATGTCTTCATTTCAATTGCCAAAGC |
| sod_R | CCTCGAGCTAATGTTTTTTTGATTGAATTGCTTGT |
