# Supplementary figures and images for: The interaction between flagellin and the glycosphingolipid Gb3 on host cells contributes to Bacillus cereus acute infection
Source: Virulence. 2020 Jun 7;11(1):769–80. doi: 10.1080/21505594.2020.1773077 (PMC7567440; doi:10.1080/21505594.2020.1773077)

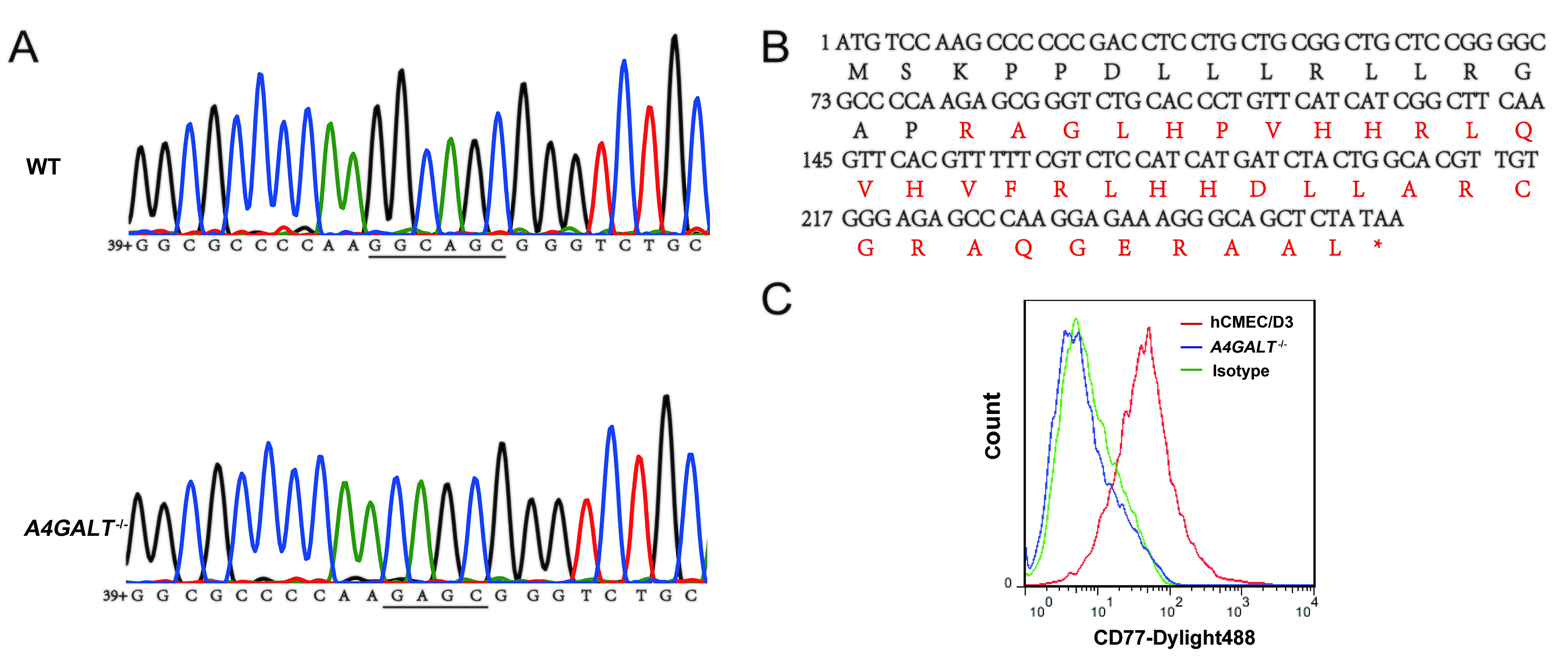

Supplement: Supplemental Material [file KVIR_A_1773077_SM2623.zip › Figure S1.tif]
